# The risk of myelodysplastic syndrome and acute myeloid leukemia by metformin use and type 2 diabetes status – a Danish nation-wide cohort study

**DOI:** 10.2340/1651-226X.2025.44082

**Published:** 2025-07-02

**Authors:** Umar Aziz

**Affiliations:** Department of Medicine, Jinnah Sindh Medical University, Karachi, Pakistan

**Keywords:** diabetes mellitus, type 2, metformin, myelodysplastic, syndromes, leukemia, myeloid, acute epidemiologic studies, biomarkers

To the Editor,

I recently came across the study by Rotbain et al. exploring the relationship between metformin use in people with type 2 diabetes (T2D) and their risk of developing myelodysplastic syndrome (MDS) or acute myeloid leukemia (AML) [1]. I thought the study was well designed, especially in its use of national registry data, and I appreciate the authors tackling such a nuanced area. That said, I would like to offer a few reflections that might be useful for future research on this topic.

First, the way the study defines metformin exposure, based completely on prescription records, could introduce some uncertainty. Just because a prescription is filled does not always mean the medication is taken as prescribed. Real-world adherence to metformin can vary quite a lot. In fact, according to research by Alfian and colleagues, about one-third of patients might not take their medications as prescribed [2]. This could dilute any real link between metformin and cancer risk. Using data from pharmacy refills or even patient self-reports might offer a clearer picture in future analyses.

I also wondered about how the study grouped people who were not taking metformin. That category probably includes patients who stopped metformin for all sorts of reasons; some might have had side effects, others because their diabetes got worse or due to other health problems. Each of these situations could carry its own risk for cancer, separate from any effect of metformin. As pointed out in a review by Khunti et al., stopping diabetes medications is often associated with disease progression or side effects [3], factors that make result interpretation more difficult. It might be helpful in future studies to break down this group further or adjust more directly for how severe the diabetes is (using measures like HbA1c or disease duration).

Another factor that stood out to me was obesity. While the study adjusted for comorbidities using the Nordic Multimorbidity Index, it did not account for obesity, which is a major risk factor for both T2D and myeloid cancers. Wang et al. showed in a meta-analysis that higher body mass index (BMI) increases the risk for both MDS and AML [4]. Including body weight or waist circumference in the analysis could help tease apart whether the increased cancer risk comes from diabetes itself or from metabolic issues tied to obesity.

Lastly, the study lacked data on crucial inflammatory and metabolic biomarkers such as C- reactive protein (CRP), insulin resistance indicators, or lipid profiles. These markers are pretty central to understanding both T2D and the risk of blood cancers such as MDS and AML. We know that chronic inflammation and metabolic disturbances play a big role in how myeloid neoplasms develop, and they could also influence how metformin works in the body. Without being able to adjust for these biomarkers, there is still a real chance that some confounding is present, which might make it harder to see the true relationship between diabetes, metformin, and cancer risk. In fact, recent studies have shown that higher levels of inflammatory markers can independently predict who will go on to develop MDS or AML [5]. I think that in the future, linking registry data with lab results or biobank samples could really help us get a clearer picture of how metabolic health and inflammation are tied to cancer risk, especially in large-scale studies like this one from Denmark.

I would like to thank the authors for their valuable contribution. As we look for ways to prevent myeloid cancers in high-risk populations, refining these approaches could help us better understand metformin’s true potential.

## Data Availability

No datasets were generated or analyzed during the current study.
